# Lack of sexual dimorphism on the inhibitory effect of alcohol on muscle protein synthesis in rats under basal conditions and after anabolic stimulation

**DOI:** 10.14814/phy2.13929

**Published:** 2018-12-04

**Authors:** Charles H. Lang

**Affiliations:** ^1^ Department of Cellular and Molecular Physiology Penn State College of Medicine Hershey Pennsylvania

**Keywords:** Blood alcohol levels, estrus cycle, muscle protein synthesis, S6K1, sex differences

## Abstract

Previous studies indicate women have a higher blood alcohol (i.e., ethanol) and acetaldehyde concentration after consuming an equivalent amount of alcohol, and that women are more susceptible to the long‐term negative health effects of alcohol. However, there is a paucity of data pertaining to whether there is a sexual dimorphic response in skeletal muscle to alcohol. Adult male and female Sprague–Dawley rats were used and the primary endpoint was in vivo determined muscle (gastrocnemius) protein synthesis (MPS). The initial study indicated MPS did not differ in female rats during proestrus, estrus, metestrus, or diestrus; hence, subsequent studies used female rats irrespective of estrus cycle phase. There was no difference in MPS between male and female rats under basal fasted conditions, and the time‐ and dose‐responsiveness of both groups to the inhibitory effect of acute alcohol did not differ. The ability of alcohol to suppress MPS was comparable in male and female rats pretreated with alcohol dehydrogenase inhibitor 4‐methylpyrazol. Chronic alcohol feeding for 6 weeks decreased MPS in male but not in female rats; however, MPS was reduced in both sexes at 14 weeks. Finally, oral gavage of leucine increased MPS similarly in male and female rats and chronic alcohol feeding for 14 weeks prevented the anabolic effect in both sexes. These data suggest normal fluctuations in ovarian hormones do not significantly alter MPS in female rats, and that there is no sexual dimorphic response to the effects of acute alcohol intoxication on MPS. While chronic alcohol consumption appeared to decrease MPS at an early time point in male compared to female rats, there was no sex difference in the suppressive effect of alcohol at a later time point. Overall, these data do not support the prevailing belief that females are more susceptible than males to alcohol's catabolic effect on MPS.

## Introduction

While there is a higher prevalence of men consuming alcohol (i.e., ethanol) than women, this gap appears to be narrowing (Breslow et al. 2017). Furthermore, women absorb and metabolize alcohol differently than men, and women are often reported to achieve higher blood alcohol levels (BAL) than men when both consume an equivalent amount of alcohol (Frezza et al. 1990). Moreover, the prevailing circulating concentration of acetaldehyde, a potentially toxic metabolite of alcohol metabolism, is also higher in women than men and can vary with menstrual cycle phase (Eriksson et al. 1996). As a result of these and other differences, the consensus of current research suggests that women are relatively more susceptible to alcohol‐related organ damage, especially related to damage to the liver (Tuyns and Pequignot 1984), brain (Devaud et al. 2003), and heart (Urbano‐Marquez et al. 1995).

Sustained and excessive alcohol consumption also produces biochemical and morphological changes in skeletal muscle that translate into a loss of muscle protein and lean body mass (Thapaliya et al. 2014; Steiner and Lang 2015). The prevalence of this alcoholic skeletal muscle myopathy has been estimated at between 45% and 70% (Preedy and Peters 1990). Specifically, both acute alcohol intoxication and chronic alcohol feeding in rats have been demonstrated to decrease the synthesis of both myofibrillar and sarcoplasmic proteins particularly in muscles with a predominance of type II fast‐twitch fibers (Lang et al. 1999; Reilly et al. 2000), that ultimately, decreases contractile function and muscle strength. In large part, the alcohol‐induced decrease in muscle protein synthesis (MPS) appears as a direct result of inhibition of mTOR (mechanistic target of rapamycin) kinase activity that limits the initiation phase of mRNA translation (Steiner and Lang 2015).

Seminal work in the area of sex differences related to the effect of alcohol on skeletal muscle myopathy has been reported (Urbano‐Marquez et al. 1995). In this clinical study, men and women exhibited a similar degree of myopathy (e.g., muscle weakness) despite women having a 60% lower mean lifetime dose of alcohol than men. In contrast, the alcohol‐induced decrease in cross‐section area of type II muscle fibers was similar in men and women (Martin et al. 1985). Hence, it remains unsettled whether the alcohol‐induced changes in muscle protein balance are impacted by sex/gender. While sex‐related differences in selected individual proteins within muscle have been reported in rats (Hunter et al. 2003; Nakahara et al. 2003, 2006), there has not been a systematic investigation of the impact of alcohol on MPS in male and female rats. Therefore, the purpose of the present study was to determine whether there is a sexually dimorphic response in MPS to acute alcohol intoxication or chronic alcohol consumption in rats.

## Materials and Methods

The experimental protocols described below were performed in accordance with the National Institutes of Health (NIH) guidelines for the use of animals and were approved by the Institutional Animal Care and Use Committee of The Pennsylvania State University College of Medicine (#46587).

### Estrus cycle

An initial study was performed to determine whether MPS was dependent on the estrus cycle phase. Vaginal smears were performed daily on female rats and rats were used only after exhibiting at least two consecutive 4‐day estrous cycles. Only those animals exhibiting normal estrous cyclicity, verified by vaginal smears, were used in this study. For all subsequently described studies described below, female rats were selected independent of estrous cycle phase.

### Acute alcohol intoxication

For this series of experiments, 7‐week old pathogen‐free male and female Sprague–Dawley rats (Crl:CD(SD); strain, Charles River Breeding Laboratories, Cambridge, MA) were used. Male and female rats were housed separately, acclimated for approximately 1 week in a controlled environment (22 ± 1°C; 30–70% humidity, 12 h:12 h light/dark cycle), and provided commercial laboratory food (8604 diet; percent calories from protein 32%, from fat 14%, and from carbohydrates 54%; Envigo Teklad, Boston, MA) and water ad libitum before the start of the study. On the day of the study, the average body weight for male rats was 265 ± 12 g while female rats of the same age weighed 223 ± 11 g. After an overnight fast, rats were randomized into two groups; the alcohol‐treated group was orally gavaged with one of three doses of ethanol (20, 50, or 75 mmol/kg body weight), while the control group was administered an equal volume of 0.9% (wt/vol) sterile saline. The highest dose of ethanol was selected because preliminary studies indicated it maximally decreased MPS. The two lower ethanol doses were selected based on previous studies which reported that the BAL was reduced approximately 50% and 90% at 1 h, respectively, compared to the BAL present in the high‐dose ethanol group. At the conclusion of this and subsequent studies, rats were anesthetized with isoflurane (3–5% in oxygen) and a midline laparotomy performed to expose the inferior vena cava for blood collection into a heparinized syringe. Thereafter, the gastrocnemius was excised and frozen between metal plates to the temperature of liquid nitrogen.

Additional studies were performed in overnight fasted male and female rats in which a maximal suppressive dose of ethanol (75 mmol/kg) was administered and the responsiveness of muscle toward insulin‐like growth factor (IGF)‐I was determined at various time points (i.e., 1, 4, 8, and 24 h) after the administration of alcohol. In these studies, rats were injected intravenously (IV) with IGF‐I (25 nmol/kg BW) or an equivalent volume of isotonic saline. This dose of IGF‐I was selected because it produces robust IGF‐I receptor phosphorylation but only a negligible increase in insulin receptor phosphorylation (Lang et al. 2004). The gastrocnemius muscle was collected 20 min after hormone injection, a point previously determined to represent the optimal time for the assessment of mTOR complex 1 (mTORC1) activity (Lang et al. 2004).

A final series of acute alcohol experiments were performed using male and female rats that were pretreated with the alcohol dehydrogenase (ADH) inhibitor 4‐methylpyrazole (4‐MP; 8 mg/kg, Sigma, St. Louis, MO)., at a dose previously demonstrated to be effective (Lang et al. 2004).

### Chronic alcohol consumption

Sprague–Dawley rats were about 5 weeks of age and acclimated in‐house for approximately 1 week during which time they were provided standard rodent chow as above. Thereafter, two rats of the same sex were housed per cage (males = 172 ± 9 g; females 153 ± 8 g) and fed a Lieber‐DeCarli ethanol‐containing liquid diet where ethanol‐derived calories were increased stepwise from 12% to 36% of total energy during first 2 weeks (Bioserv, Frenchtown, NJ). Control‐fed rats received an isonitrogenous isocaloric liquid diet containing maltose dextrin instead of ethanol, and the volume provided was the average consumed by ethanol‐fed rats of the same sex on the previous day. Alcohol‐ and control‐fed rats were provided liquid diet for either 6 or 14 weeks. In a separate group of rats, the anabolic effect of leucine was assessed. Briefly, control‐ and alcohol‐fed rats consumed the appropriate liquid diet for 14 weeks, where fasted overnight, and then administered either saline (0.155 mol/L) or 1.35 g/kg BW leucine (prepared as 54.0 g/L of l‐amino acid in distilled water) by oral gavage. Muscle and blood were collected 1 h after administration of leucine. This dose of leucine was selected because it is equivalent to the total amount of leucine normally consumed in a 24‐h period by rats of this age and strain (Lang et al. 2003).

### Plasma measurements

Plasma alcohol concentrations were determined at the time of euthanization (Analox Instruments, Lunenburg, MA). Plasma insulin concentrations were analyzed using a commercial ELISA kit for rat insulin (ALPCO, Windham, NH). Plasma leucine concentrations were determined using reversed‐phase HPLC after precolumn derivatization of amino acids with o‐phthaldialdehyde as described previously (Lang et al. 2009).

### In vivo protein synthesis

The rate of in vivo global mixed protein synthesis was determined by using a) the flooding‐dose method in which ^3^H‐phenylalanine incorporation was quantitated (Vary and Lang 2008), or (b) the nonisotopic SUnSET method where puromycin incorporation into muscle protein was determined (Goodman and Hornberger 2013). Although two methods were used as our laboratory gradually transitioned from the radioactive to nonradioactive method, only a single method was used for each experimental series containing male and female rats. Protein synthesis was determined using the flooding‐dose technique performed exactly as described (Vary and Lang 2008). Rats were injected via the tail vein with [^3^H]‐L‐phenylalanine (Phe; 150 mmol/L, 30 *μ*Ci/mL; 1 mL/100 g BW). Rats were anesthetized after 15 min with isoflurane (3–5% in oxygen). Blood was collected from the abdominal aorta and skeletal muscle was freeze‐clamped to the temperature of liquid nitrogen. All samples were than stored at −70°C. Blood and tissues were processed exactly as described (Vary and Lang 2008). The specific radioactivity of the plasma Phe was measured by high‐performance liquid chromatography (HPLC) analysis of supernatant from trichloroacetic acid (TCA) extracts of plasma. In other experiments, protein synthesis was determined by the SUnSET method exactly as described (Steiner et al. 2016). Rats were injected via the tail vein with puromycin (0.04 *μ*mol/g) 15 min prior to euthanasia. Animals were thereafter treated the same as described above. Muscle obtained from these rats was homogenized in ice‐cold homogenization buffer consisting of (in mmol/L) 48.3 HEPES (pH 7.4), 4 EGTA, 10 EDTA, 15 sodium pyrophosphate, 100 β‐glycerophosphate, 25 sodium fluoride, 5 sodium vanadate, 0.1% Triton X‐100, and 1 *μ*L/mL protease inhibitor. The amount of protein in each sample was determined using BioRad protein assay kit (Bio‐Rad; Hercules, CA). Western blotting was performed on equal amounts of total protein per sample using an antipuromycin antibody for immunological detection of puromycin‐labeled peptides (Kerafast, Boston, MA).

### Western blotting

Homogenates were clarified by centrifugation and mixed with 2× Laemmli SDS sample buffer. Equal amounts of protein per sample were subjected to electrophoresis on 12% SDS‐PAGE gels for total and phosphorylated S6K1 (Thr389) (Cell Signaling Technology, Danvers, MA). Proteins were transferred onto polyvinylidene fluoride (PVDF; Immobilon P) membranes and incubated with a primary antibody overnight at 4°C. The blots were developed using enhanced chemiluminescence western blotting reagents and then exposed to X‐ray film in a cassette equipped with a DuPont Lightening Plus intensifying screen; films were scanned and analyzed using NIH Image 1.6 software.

### Statistics

Values were presented as means ± SEM for the number of rats per group as indicated in the figures. The data were analyzed using two‐way analysis of variance with post hoc Student–Newman–Keuls test to determine significant differences among the four experimental groups. Differences were considered significant when *P* < 0.05.

## Results

### Effect of estrus cycle phase

**Figure 1:**
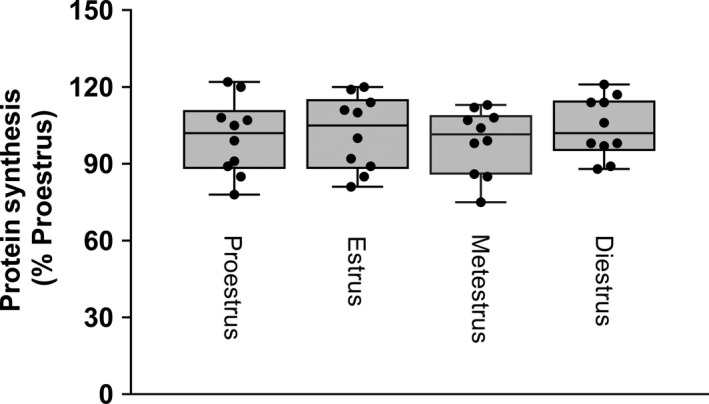
In vivo ‐determined muscle protein synthesis assessed during the four phases of the estrus cycle. Regularly cycling female rats were staged using vaginal smears and protein synthesis determined using ^3^H‐phenylalane incorporation into muscle protein. Values are means with the minimum and maximum values indicated by upper and lower bars, respectively; *n* = 10 per group with individual values plotted for each group. There were no statistical differences between groups based on one‐way ANOVA with Student–Newman–Keuls (SNK) post hoc test.

Muscle protein synthesis was determined in regularly cycling rats during all four phases of the estrus cycle. Mixed MPS did not differ between the phases of the estrus cycle (Fig. 1). As a result of this finding, subsequent studies did not determine estrus cycle phase and female rats were randomly assigned between various experimental groups.

### Acute alcohol administration

**Figure 2:**
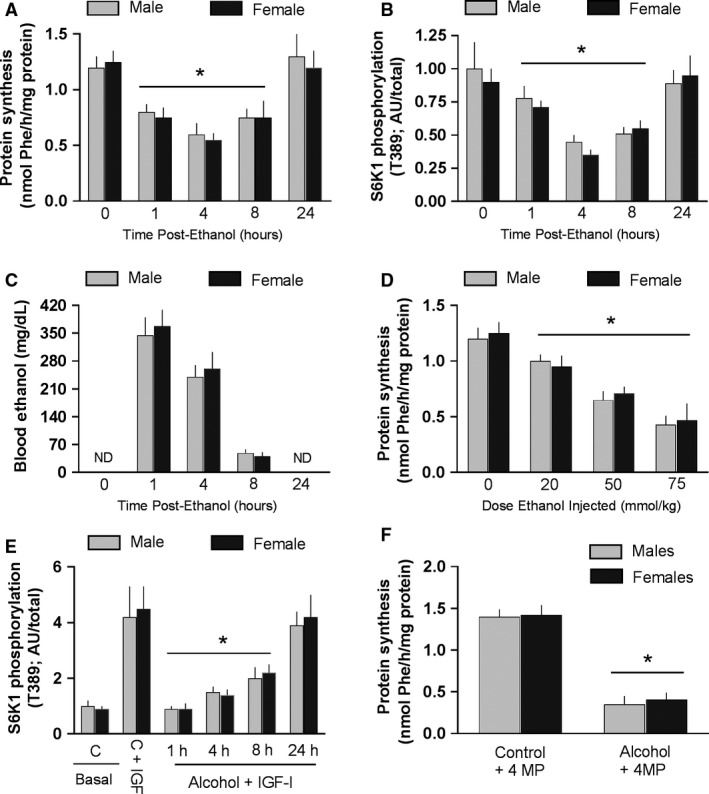
Muscle protein synthesis in male and female rats receiving acute alcohol administration. Values are means ± SEM. *P < 0.05 compared to respective basal control value. ND = not detectable. There were no statistical differences between male and female rats for any parameter examined. Panels A, B and C, protein synthesis, T389‐phosphorylation of S6K1, and the blood alcohol concentration determined at various time points (1–24 h) after oral gavage of 75 mmol/kg of alcohol. Sample size equals 8–10 rats per time point for both male and female rats. Panel D, protein synthesis determined at 4 h in response to various doses of alcohol. Sample size equals 7–8 rats per dose of alcohol for both male and female rats. Panel E, S6K1 phosphorylation (surrogate marker of protein synthesis) in response to IGF‐I under control conditions and at various times after or gavage of alcohol. Sample size equals 8–9 rats per treatment group for each male and female rat. Panel F, effect of 4‐methylpyrazole (MP) inhibition of alcohol dehydrogenase activity prior to oral gavage of alcohol (75 mmol/kg); *n* = 6 rats in each group. Data in panels A, B, D, E, and F were analyzed by one‐way ANOVA with Dunnett's post hoc test against the sex‐specific Time = 0 control value. Additionally, differences between mean values for male versus female rats at the same time point or ethanol dose were compared using a pooled Student's *t*‐test.

There was no difference in the absolute rate of protein synthesis in muscle between male and female rats under basal conditions (e.g., time 0; Fig. 2A). Oral administration of a high dose of alcohol [75 mmol/kg body weight (BW)] decreased MPS by 1 h, this decrease persisted for at least 8 h after alcohol, and the inhibitory effect of alcohol did not differ between male and female rats (Fig. 2A). Acute alcohol produced a similar temporal decrease in S6K1 T389 phosphorylation in muscle and the decrease in this surrogate marker of mTORC1 kinase activity (Steiner and Lang 2015) did not differ between male and female rats (Fig. 2B). Furthermore, the BAL was increased at 1–8 h after alcohol, but there was no sex difference in the BAL determined at these time points (Fig. 2C). To determine whether male and female rats might have a different sensitivity to the suppressive effects of alcohol, a dose–response study was performed. However, there was no sex difference in the ability of lower doses of alcohol to suppress MPS in male and female rats (Fig. 2D). Alcohol can also produce an anabolic resistance to the stimulatory effects of nutrients (e.g., leucine) and hormones (e.g., insulin and IGF‐I) (Lang et al. 2004). However, the ability to suppress S6K1 phosphorylation did not differ between male and female rats (Fig. 2E). Finally, 4‐MP was injected prior to alcohol administration to prevent the oxidative metabolism of ethanol to acetaldehyde. Again, there was no difference in the alcohol‐induced decrease in MPS between male and female rats in the presence of this inhibitor (Fig. 2F).

### Chronic alcohol feeding

**Figure 3:**
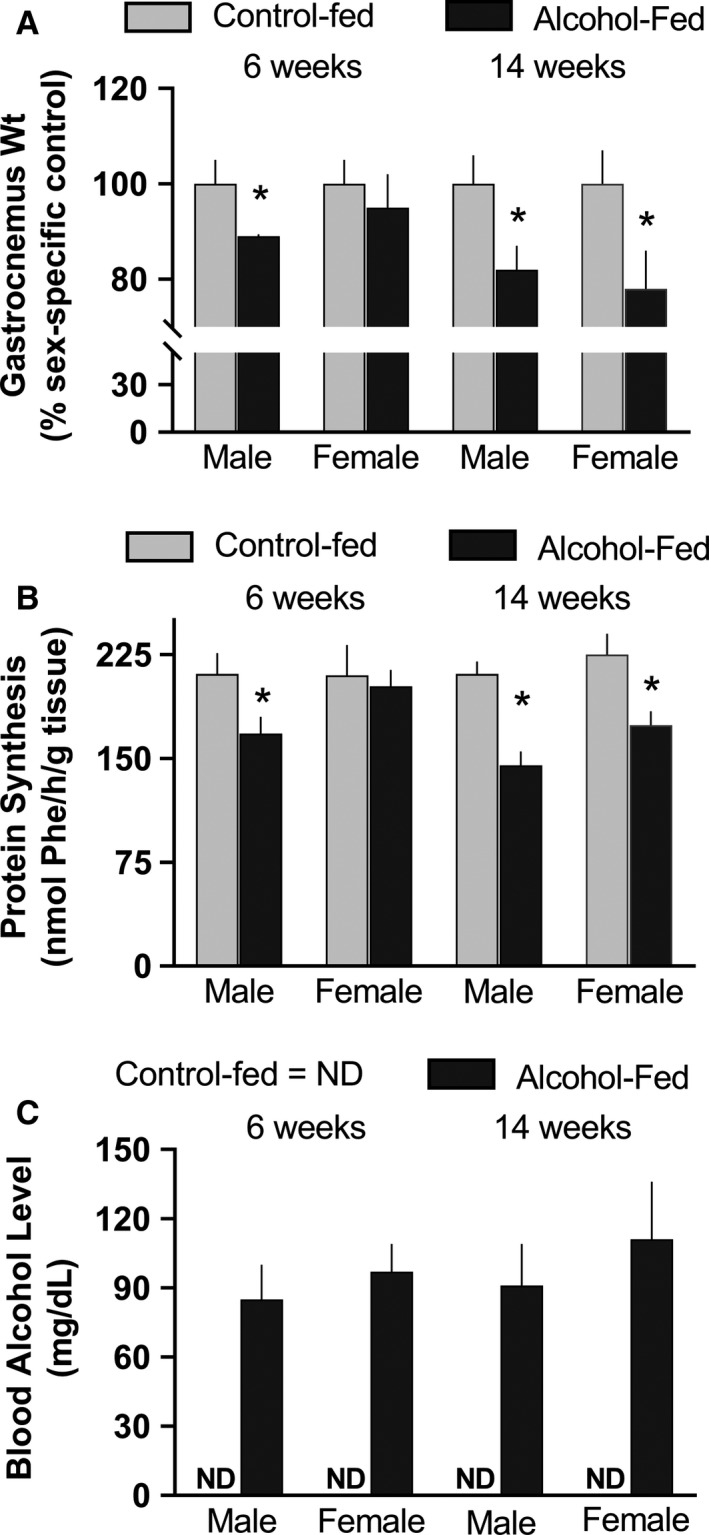
Muscle protein weight and synthesis in alcohol‐fed and control‐fed male and female rats after 6 and 14 weeks of feeding. Values are means ± SEM; *n* = 9–11 for each group for each male and female rat. Panels A and B, *P < 0.05 compared to time‐matched value from male rats. Panel C, blood alcohol level (BAL) in control‐fed and alcohol‐fed mice; ND = not detectable. There was no statistical difference between BAL values between groups. For gastrocnemius weight and protein synthesis, differences between male and female rats at a given time point were compared using a pooled Student's *t*‐test.

Male and female rats were fed a complete alcohol‐containing diet or were pair‐fed for either 6 weeks or 14 weeks. Male rats demonstrated a reduction in muscle mass and protein synthesis after 6 weeks, a response that was not observed in female rats (Fig. 3A and B). However, a comparable decrease in muscle mass and protein synthesis was detected in both male and female rats at 14 weeks. This temporal difference to chronic alcohol consumption between male and female rats could not be explained by a difference in the circulating concentration of alcohol, as the BAL did not differ between groups (Fig. 3C). The BAL in control‐fed rats was below the limit of detection.

**Figure 4:**
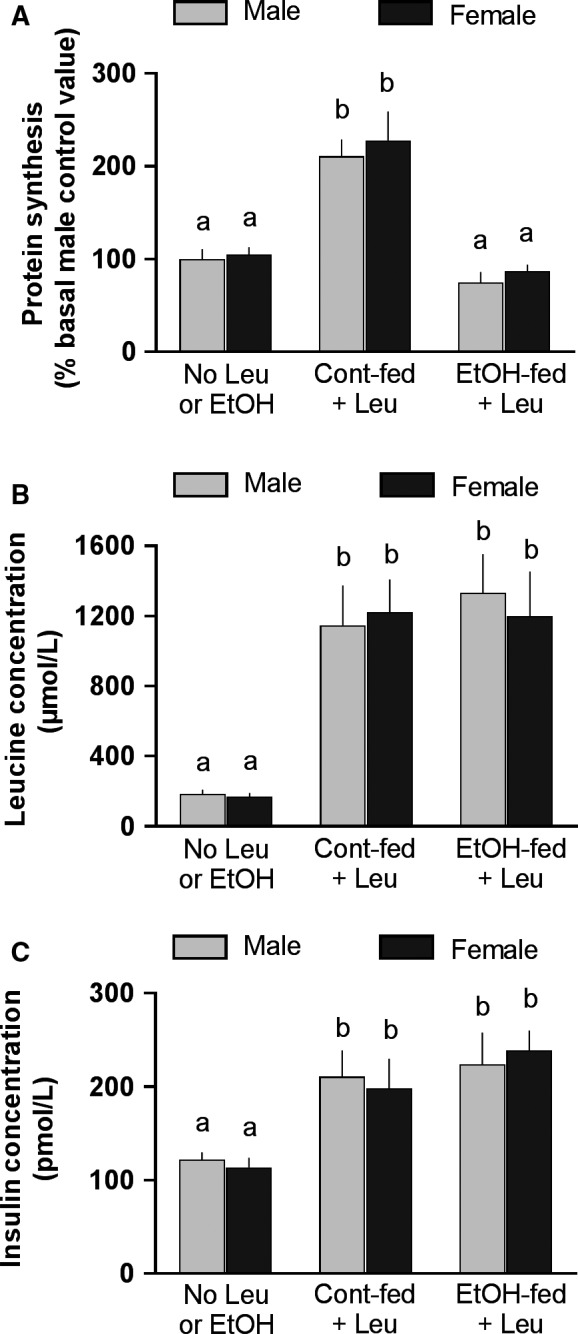
Effect of oral leucine (Leu) gavage on muscle protein synthesis, and plasma leucine and insulin concentrations in control‐ and alcohol‐fed rats. Both male and female rats were fed an alcohol‐containing liquid diet or isocaloric isonitrogenous diet for 14 weeks, then fasted overnight, and administrated an oral gavage of a maximally stimulating dose of Leu (1.35 g/kg body weight) and gastrocnemius and blood collected 1 h thereafter. Values are means ± SEM; *n* = 7–8 for each treatment group for each male and female rat. Values with different letters are statistically different (*P* < 0.05) from each. Data were analyzed by one‐way ANOVA with Dunnett's post hoc test against the sex‐specific control value (e.g., no leucine or EtOH group).

Data in Figure 4A indicate that both male and female control‐fed (no alcohol) rats demonstrated a significant and comparable increase in MPS in response to an oral gavage of a maximally stimulating dose of leucine. Moreover, MPS was equally suppressed by 14 weeks of alcohol feeding in male and female rats. There were no differences in the concentrations of two key regulators of MPS, leucine (Fig. 4B) and insulin (Fig. 4C), between male and female rats under basal fasted conditions, or in response to the leucine gavage alone in either the pair‐fed control or the alcohol‐fed rats.

## Discussion

The purpose of the present study was to determine whether there was a sexual dimorphic response to alcohol‐induced changes in MPS in rats. An initial study was performed to determine MPS in female rats during the four stages of the estrus cycle, during which there are dynamic changes in the physiological concentrations of estrogen and progesterone (Fuentes et al. 2018). Previous reports indicate that MPS is increased after ovariectomy, and the addition of estrogen or progesterone to these rats returned the synthetic rate back to control values (Toth et al. 2001). These data suggest that high physiological levels of female sex hormones may normally inhibit MPS. One limitation of this study is that gondal steroid hormones were not assessed in either female or male rats, and therefore the potential role of alcohol‐induced changes in the circulating concentrations of one or more these hormonal regulators could not be assessed relative to changes in MPS. However, our data clearly indicate there was no discernable effect of estrus cycle phase on the rate of mixed protein synthesis in gastrocnemius. These data are consistent with an earlier report in women indicating no difference in MPS when examined during the follicular phase (e.g., relatively low estradiol and progesterone) and luteal phase (relatively high estradiol and progesterone) of the menstrual cycle (Miller et al. 2006). Hence, as a result of this observation, female rats used in subsequent studies were randomly assigned to various treatment groups irrespective of estrus cycle phase.

The next series of experiments were performed to determine whether there was a sexually dimorphic response in MPS to acute alcohol intoxication alone or in combination with the anabolic hormone IGF‐I. First, in these acute studies it is noteworthy to observe that the blood alcohol concentration did not differ between male and female rats, and this finding is consistent with the lack of a sex difference in the area under the curve (AUC) for the clearance of alcohol as well as the lack of a difference in the AUC in difference phases of the estrus cycle (Kelly et al. 1987). Second, our data indicate that there is no difference in MPS or the relative abundance of T389‐phosphorylated S6K1 between male and female rats in the fasted condition. These data are consistent with previous reports in humans where both MPS and mTOR activity (i.e., S6K1 and 4E‐BP1 phosphorylation) were compared in men and women (Markofski et al. 2015), and there was no sex effect on rates on *whole‐body* protein synthesis (Volpi et al. 1998). While these data indicate no sex difference for MPS, they should not be extrapolated to individual muscle proteins that may be differentially expressed in male and female rats (Nakahara et al. 2003, 2006). Third, the time‐ and dose‐dependent inhibitory effect of alcohol on MPS also did not differ between male and female rats. Fourth, the phosphorylation state of S6K1 (and by inference protein synthesis) did not differ between male and female rats in the absence or over time after acute alcohol administration. And finally, the alcohol‐induced decrease in MPS was comparable between male and female rats where the production of acetaldehyde and other potentially toxic ethanol metabolites were largely prevented by 4‐MP inhibition of alcohol dehydrogenase. These latter results suggest that the direct effect of alcohol on MPS, independent of any potential difference in the generation of oxidative metabolites, does not differ between male and female rats. Collectively, these data fail to demonstrate any sexually dimorphic response in MPS to acute alcohol administration.

Contrary to expectations, male rats consuming a nutritionally complete liquid diet demonstrated a reduction in MPS at an earlier time point than female rats (6 vs. 14 weeks duration). This earlier decrease in MPS in male rats was not associated with a difference in the prevailing BAL. Similar BAL between male and female rats consuming the liquid diet is consistent with previous studies (Kennedy et al. 2002; Piano et al. 2005). Moreover, the anabolic effect of leucine was comparable in male and female rats under basal conditions, and there was no sex difference in the ability of chronic alcohol consumption to inhibit leucine‐induced increases in MPS. Leucine was used in this study as this branched‐chain amino acid is a major regulator of mTORC1 and MPS (Anthony et al. 2000). These results are consistent with the similar increase in the plasma concentration of insulin and leucine in response to leucine gavage in both sexes.

As indicated above, muscle mass is proportional to total protein content which is determined both by the rate of protein synthesis as well as the rate of protein degradation. While this study focused on the synthetic side of the protein balance equation, it remains possible that one or more of the various proteolytic pathways in muscle may show a sexual dimorphic response to alcohol and/or nutrient or hormonal stimulation. However, as recently reviewed, overall there is little definitive evidence that consistently demonstrates that alcohol alters rates of protein degradation in skeletal muscle (Steiner and Lang 2015).

Overall, these data do not support the general perception in the literature that females are more susceptible than males to alcoholic skeletal muscle myopathy, which appears to be primarily founded on an earlier study that reported women had the same reduction in muscle strength as men despite a lower lifetime consumption of alcohol (Urbano‐Marquez et al. 1995). However, the data in this paper also reveal that there was no sex difference in the slope of the linear relationship between total lifetime dose of alcohol and muscle strength. Such data suggest both sexes exhibit a comparable decrement in strength with an increased consumption of alcohol and the earlier appearance of the muscle weakness in women is a result of their lower basal muscle mass. Consistent with this conclusion, the data from Martin et al. (1985) indicate a relatively similar alcohol‐induced decrease in type II fiber cross‐section area between men and women. As alcohol‐induced skeletal muscle myopathy is clinically relevant in both men and women, and the current data indicate no marked sex differences in MPS, there is strong justification for the inclusion of both male and female rats in future studies examining the underlying mechanisms. This conclusion supports recent efforts by the National Institutes of Health to consider sex as a biological variable in preclinical studies in general (Clayton 2016) and in alcohol‐focused studies in particular (Guizzetti et al. 2016).

## Conflict of Interest

The author declares no conflict of interest.
